# Preadmission predictors of graduation success from a physical therapy education program in the United States

**DOI:** 10.3352/jeehp.2019.16.5

**Published:** 2019-02-26

**Authors:** Gretchen Roman, Matthew Paul Buman

**Affiliations:** 1 Physical Therapy, Midwestern University, Glendale, AZ, USA; 2 College of Health Solutions, Arizona State University, Phoenix, AZ, USA; Hallym University, Korea

**Keywords:** Education, Physical therapy, School admission criteria, United States

## Abstract

**Purpose:**

The field of physical therapy education is seeking an evidence-based approach for admitting qualified applicants, as previous research has assessed various outcomes, impeding practical application. This study was conducted to identify preadmission criteria predictive of graduation success.

**Methods:**

Data from the 2013–2016 graduating cohorts (n=149) were collected. Predictors included verbal Graduate Record Examination rank percentile (VGRE%), quantitative GRE rank percentile, analytical GRE rank percentile, the admissions interview, precumulative science grade point average (SGPA), precumulative grade point average (UGPA), and a reflective essay. The National Physical Therapy Examination (NPTE) and grade point average at the time of graduation (GGPA) were used as measures of graduation success. Two separate mixed-effects models determined the associations of preadmission predictors with NPTE performance and GGPA.

**Results:**

The NPTE model fit comparison showed significant results (degrees of freedom [df]=10, P=0.001), decreasing within-cohort variance by 59.5%. NPTE performance was associated with GGPA (β=125.21, P=0.001), and VGRE%, the interview, the essay, and GGPA (P≤0.001) impacted the model fit. The GGPA model fit comparison did not show significant results (df=8, P=0.56), decreasing within-cohort variance by 16.4%. The GGPA was associated with the interview (β=0.02, P=0.04) and UGPA (β=0.25, P=0.04), and VGRE%, the interview, UGPA, and the essay (P≤0.02) impacted model fit.

**Conclusion:**

In our findings, GGPA predicted NPTE performance, and the interview and UGPA predicted GGPA. Unlike past evidence, SGPA showed no predictive power. The essay and VGRE% warrant attention because of their influence on model fit. We recommend that admissions ranking matrices place a greater weight on the interview, UGPA, VGRE%, and the essay.

## Introduction

Multiple preadmission predictors have been studied by physical therapy (PT) and PT assistant education programs. Such predictors have included age [1], gender [1,2], entry-level degree [1,3], Graduate Record Examination (GRE) [2], prerequisite grade point average (GPA) [1], precumulative science grade point average (SGPA) [2,3], precumulative grade point average (UGPA) [1-3], essays [2], and letters of recommendation [2]. These preadmission predictors have been studied with reference to a variety of outcomes, including GPA after the first professional year [1], admission [2], and National PT Examination (NPTE) performance [3].

Past evidence has demonstrated certain associations between preadmission predictors and various outcomes. Jones et al. [4] in 2014 reviewed the admissions processes of PT and physician assistant programs and noted UGPA to be the best predictor of academic success. Ruscingno et al. [1] in 2010 found that UGPA was correlated with the basic sciences GPA after the first professional year [1]. Nuciforo et al. [2] in 2014 found that SGPA was the strongest predictor of PT program admission. In an analysis of PT programs at the University of Tennessee at Chattanooga and University of North Dakota, no difference was found in the NPTE performance of individuals with differing entry-level degrees, defined as 3 versus 4 years of preprofessional coursework [3]. The field of PT education is still seeking an evidence-based approach for admitting qualified applicants. A considerable amount of research exists; however, previous studies analyzed multiple distinct outcomes, impeding practical application. This comprehensive study will add to the available literature by using multiple preadmission predictors, with NPTE performance and GPA at the time of graduation (GGPA) as outcomes of graduation success.

The aim of this study was to determine which previously identified preadmission criteria were predictors of graduation success from a private PT program in the southwestern United States by using a rigorous statistical analysis representative of the multi-faceted admissions process. We hypothesized that verbal, quantitative, and analytical GRE rank percentile (VGRE%, QGRE%, and AGRE%), the admissions interview, SGPA, UGPA, and the reflective essay would all be predictors of NPTE performance and GGPA.

## Methods

### Ethical statement

This study was approved by the Institutional Review Board at Midwestern University in Glendale, AZ, USA (#970).

### Study design

This was a retrospective cohort study.

### Materials and/or subjects

Data from admission to graduation of the graduating cohorts of 2013–2016 (n=149) at a 3-year graduate-level Doctor of PT program were collected. Seventy percent of the students from 2013, 91% from 2014, 87% from 2015, and 84% from 2016 released the data necessary for this study’s statistical analyses.

### Technical information

Demographics at admission, preadmission criteria, and interview and essay scores were compiled via records from the Office of Admissions. The GGPA was obtained from the Registrar’s Office and the released NPTE scores were provided by the PT program.

### Statistics

#### Independent variables

Preadmission predictors included VGRE%, QGRE%, AGRE%, the admissions interview, SGPA, UGPA, and the reflective essay. The applicants were scored by 2 raters at the in-person, on-campus admissions interview. Scores were based on a rubric with 7 subscales (4 points per subscale for a total of 28 points), including appearance, body language, communication, experience, knowledge of the PT profession, teamwork/interpersonal skills, and resilience/planning/organization. Reflective essays (composed during the interview) were scored by 1 rater (a PT Admissions Committee member) and scores were based on a rubric with 2 subscales (4 points per subscale, for a total of 8 points) including ethics/integrity and problem solving/critical thinking.

#### Dependent variables

The NPTE score (out of 800 points) served as the outcome of graduation success for the initial analysis, and the GGPA served as the outcome for the secondary analysis.

With statistical significance set at α<0.05, all statistical analyses were conducted using IBM SPSS ver. 24.0 (IBM Corp., Armonk, NY, USA). Descriptive statistics provided an overview of the mean values of the outcome variables. All models were assessed for normality. A 2-way random intraclass correlation coefficient (ICC) for consistency was selected to measure the interrater reliability of the interview scores. Two separate mixed-effects models, nested by graduating cohort, were used to determine the associations of preadmission predictors with NPTE performance and GGPA between and within graduating cohorts. Correlated predictors were residualized because of concerns regarding multicollinearity and subsequent effects on beta coefficient estimates. In the initial analysis of preadmission predictors and NPTE, step 1 involved configuring the unconditional means model (UMM). The remaining steps involved inputting VGRE% (step 2), QGRE% (step 3), AGRE% (step 4), the interview score (step 5), SGPA (step 6), UGPA (step 7), the residualized product term interaction between UGPA and SGPA (UGPA×SGPA, step 8), the essay score (step 9), GGPA (step 10), and the residualized product term interaction between UGPA and GGPA (UGPA×GGPA, step 11). For the secondary analysis of preadmission predictors and GGPA, step 1 involved configuring the UMM. The remaining steps involved inputting VGRE% (step 2), QGRE% (step 3), AGRE% (step 4), the interview score (step 5), SGPA (step 6), UGPA (step 7), UGPA×SGPA (step 8), and the essay score (step 9).

The analyses adhered to the hierarchical maximum likelihood method [5]. Only fixed effects (i.e., on average across all cohorts) were explored in the models. The model did not converge when random effects were entered, causing individual-level differences to be computed as redundant, most likely because of the limited number of cohorts in the model. Only level-one or within-individual variables, and not level-two or between-cohort variables, were explored. Explanation of variance between and within cohorts was determined by the pseudo R^2^ [5]. The negative 2-log likelihood, Akaike information criterion, and Schwarz’s Bayesian information criterion were used for model comparison.

## Results

The results for the demographics and descriptive statistics of the sample are presented in Table 1. There was a slightly greater representation of males compared to females. The mean age was similar across the graduating cohorts (range, 21 to 36 years). The sample was predominantly Caucasian, with Hispanic students representing the second largest ethnic background. Seventy-three percent of students originated from 24 states other than Arizona. The mean GGPA was similar across graduating cohorts, ranging from 2.96 to 3.95. The licensure first-time pass rate and scores on the NPTE demonstrated an upward, stabilizing trend. Overall, there were 9 first-time failures on the NPTE, and scores ranged from 515 to 800 points.

### Interrater reliability of the interview scores

Spearman correlation analysis was used to determine the associations between the interview scores of rater 1 and rater 2 (r=0.5, P=0.001). The average-measures ICC (0.7, P=0.001) and the single-measures ICC (0.5, P=0.001) were significant for 105 students out of the total sample (interview scores were not available for the entire 2013 graduating cohort, 9 members of the 2014 graduating cohort, and 4 members of the 2015 graduating cohort).

### Mixed-effects model associated with the National Physical Therapy Examination

The UMM (step 1) from the mixed-effects model for NPTE (Table 2) suggested that 97% of the total variance in NPTE was within-cohort (ICC=0.03). Step 2 revealed no associations between the main effects of VGRE% (P=0.75) with NPTE performance. Adding VGRE% in step 2 impacted model fit (P=0.001), but did not help explain the within-cohort (R^2^=-0.0034) or between-cohort (R^2^=-0.023) variance. Step 3 revealed no associations of the main effects of VGRE% and QGRE% (P≥0.15) with NPTE performance. Inputting QGRE% in step 3 did not impact model fit (P=0.17) and explained 0.6% of the within-cohort and 45.2% of the between-cohort variance. Step 4 revealed no associations of the main effects of VGRE%, QGRE%, and AGRE% (P≥0.07) with NPTE performance. Adding AGRE% in step 4 did not impact model fit (P=0.08) and explained 1.0% of the within-cohort and 89.2% of the between-cohort variance. Since QGRE% and AGRE% in the initial levels of the analysis helped to explain all the between-cohort variance, although the values were small at 3%, additional intercept values under random effects beyond step 4 were determined to be not applicable.

The remaining predictors in steps 5–11 decreased the overall within-cohort (between-person) variance. Step 5 revealed no associations of the main effects of VGRE%, QGRE%, AGRE%, and the interview (P≥0.14) with NPTE performance. Adding the interview score to step 5 impacted model fit (P=0.001) and explained 20.4% of the within-cohort variance. Step 8 conveyed no associations of the main effects of VGRE%, QGRE%, AGRE%, interview score, SGPA, UGPA, and UGPA×SGPA (P≥0.12) with NPTE performance. Adding SGPA, UGPA, and UGPA×SGPA from steps 5-8 did not impact model fit (degrees of freedom [df]=3, P=0.75) and explained an additional 1.2% of the within-cohort variance. Step 9 revealed no associations of the main effects of VGRE%, QGRE%, AGRE%, interview score, SGPA, UGPA, UGPA×SGPA, and the reflective essay (P≥0.07) with NPTE performance. Inputting the essay in step 9 impacted model fit (P=0.001) and explained 12.7% of the within-cohort variance. Lastly, step 11 revealed the only association of this initial model, between GGPA and NPTE performance (P=0.001). The other predictors remained without a significant association. For every 1-point increase in GGPA, the NPTE score increased by 125.2 points. Model fit improved from steps 9 to 11 (df=2, P=0.001) and explained 42.0% of the within-cohort variance. The model fit comparison from steps 1–11 showed significant results (df=10, P=0.001), between-cluster variance was fully explained, and within-cohort variance decreased by 59.5%.

### Mixed effects model associated with the grade point average at the time of graduation

The UMM (step 1) from the mixed-effects model for GGPA (Table 3) suggested that 100% of the total variance in GGPA was within-cohort (ICC=0.00). The initial levels of the secondary analysis (step 4) revealed no associations of the main effects of VGRE%, QGRE%, and AGRE% (P≥0.50) with GGPA. Adding AGRE% in step 4 did not impact model fit (P=0.50) and explained 0.3% of the within-cohort variance. Step 5 revealed an association between the main effects of the interview and GGPA (P=0.04), but no associations of VGRE%, QGRE%, and AGRE% (P≥0.64) with GGPA were found. Inputting the interview into step 5 impacted model fit (P=0.02) and explained 13.4% of the within-cohort variance. Step 6 again revealed an association between the interview and GGPA (P=0.04), but no associations of VGRE%, QGRE%, AGRE%, and SGPA (P≥0.54) with GGPA were found. Adding SGPA into step 6 had no impact on model fit (P=0.54) and explained 0.4% of the within-cohort variance. Step 7 revealed associations between the interview and GGPA (P=0.02) and between UGPA and GGPA (P=0.02), but no associations were found between the other predictors (P≥0.48) and GGPA. Adding UGPA in step 7 impacted model fit (P=0.02) and explained an additional 5.4% of the within-cohort variance. Associations between the interview and GGPA (P=0.02) and between UGPA and GGPA (P=0.04) remained significant in step 8, but no associations were revealed between the other predictors (P≥0.37) and GGPA. There was no impact on model fit from steps 7–8 (P=0.51) and an additional 0.4% of within-cohort variance was explained. Lastly, the association between the interview and GGPA (P=0.04) was again significant in step 9. The other predictors listed remained without a significant association (P≥0.23). For every 1-point increase of the interview score, the GGPA increased by 0.02 points. Inputting the essay scores in step 9 impacted model fit (P=0.001), but did not help to explain the within-cohort variance (R^2^=−0.007). The model fit comparison from steps 1–9 did not yield significant results (df=8, P=0.56) and within-cohort variance decreased by 16.4%.

## Discussion

Our findings suggest that GGPA predicted NPTE performance, and that the admissions interview and UGPA predicted GGPA. Overall, GGPA, the interview, and the essay best explained within-cohort variance for NPTE performance, and VGRE%, the interview, the essay, and GGPA impacted model fit. Of all the criteria tested in the initial analysis, only GGPA was associated with NPTE performance. This lone finding provided a rationale for conducting a secondary analysis with GGPA as the outcome. For GGPA, the interview and UGPA best explained within-cohort variance, and VGRE%, the interview, the essay and UGPA impacted model fit. Of all the criteria tested in the secondary analysis, the interview and UGPA were associated with GGPA. Unlike past evidence [2,3], this study did not find any significant associations between SGPA and the outcomes of interest. Since the interview helped to explain the within-cohort variance and impacted model fit for both NPTE performance and GGPA, it exhibited a significant impact. While time, expense, and implicit biases [6] serve as deterrents to hosting admissions interviews, results of this research suggest the 41% [7] of PT programs currently not requiring interviews should potentially re-consider.

This study suggests that VGRE% and the reflective essay should be considered as predictors of graduation success. Although Moneta-Koehler et al. [8] in 2017 revealed that the GRE did not predict success in biomedical graduate school, the VGRE% in this study impacted model fit for both NPTE performance and GGPA. This finding for VGRE% indicates that it remains a valid predictor. The reflective essay also warrants attention because of its impact on model fit for both NPTE performance and GGPA. This provides validity for continued use of the essay as an assessment of the reflective skills necessary for graduation success.

Although between-cohort variance for NPTE performance was fully explained by QGRE% and AGRE%, these differences were minimal. No between-cohort variance was expressed for the GGPA model. The minimal or absent between-cohort variance in the NPTE and GGPA models, respectively, confirmed the homogeneity across graduating cohorts from this PT program. Within-cohort variance explained by QGRE% and AGRE% was also minimal, indicating the lack of significance of these GRE subscales in predicting NPTE performance.

While mixed-effects modeling provides a rigorous statistical analysis representative of the multi-faceted admissions process, 37.4% of what predicted NPTE and 83.6% of what predicted GGPA remained unexplained by the multiple predictors analyzed. Other, yet to be identified, predictors of graduation success must explain the remaining variance in NPTE performance and GGPA. The authors support the movement toward more holistic admissions [9-11] and believe that the variables considered as part of that process will further explain what predicts graduation success in the field of PT education. Further analysis needs to be done on the objective measures used to quantify an applicant’s interpersonal skills, resilience, tenacity, and grit. The generalizability of these findings is limited since data were collected from only 1 program; however, 25 different home states were represented, which is a fair representation of a nationally based sample. Future collaboration with local and national PT programs will further enhance the generalizability of our findings and help inform the admissions practices of PT programs on a broader scale.

Meanwhile, the outcomes of these analyses have informed adjustments made to the weighting of each preadmission predictor within the ranking matrix used by our PT program. Out of a total of 100%, the average interview score remains a strong determinant at 25%, and the reflective essay continues to account for 10% of an applicant’s rank. Previously weighted at 15% and 25%, respectively, UGPA and SGPA now determine 25% and 15% of an applicant’s rank. The VGRE%, QGRE%, and AGRE%, which were previously weighted at 10%, 5%, and 10%, now determine 15%, 5%, and 5% of an applicant’s rank, respectively. We recommend that admissions ranking matrices designate greater weight to the admissions interview, UGPA, VGRE%, and the essay.

## Figures and Tables

**Table 1.** Demographics and descriptive statistics of the 2013–2016 graduating cohorts (n=149)

Characteristic	Year				Total
	2013	2014	2015	2016	
Total	31 (20.8)	42 (28.2)	39 (26.2)	37 (24.8)	149 (100.0)
Admission age (yr)	24.4±3.4	24.3±2.0	24.7±2.5	24.4±2.7	24.5±2.6
20–24	23 (74.2)	20 (47.6)	20 (51.3)	24 (64.9)	87 (58.4)
25–29	6 (19.4)	17 (40.5)	14 (35.9)	12 (32.4)	49 (32.9)
30–34	1 (3.2)	0	3 (7.7)	1 (2.7)	5 (3.3)
35–39	1 (3.2)	0	0	0	1 (0.7)
Unknown	0	5 (11.9)	2 (5.1)	0	7 (4.7)
Sex					
Male	15 (48.4)	21 (50.0)	28 (71.8)	19 (51.4)	83 (55.7)
Female	16 (51.6)	21 (50.0)	11 (28.2)	18 (48.6)	66 (44.3)
Ethnic background					
Caucasian	23 (74.2)	30 (71.4)	34 (87.1)	27 (73.0)	114 (76.5)
African American	1 (3.2)	1 (2.4)	0	1 (2.7)	3 (2.0)
Hispanic	4 (12.9)	2 (4.8)	1 (2.6)	6 (16.2)	13 (8.7)
Asian	0	2 (4.8)	1 (2.6)	2 (5.4)	5 (3.4)
Pacific Islander	0	1 (2.4)	0	0	1 (0.7)
Multi-racial	1 (3.2)	1 (2.4)	0	1 (2.7)	3 (2.0)
Unknown	2 (6.5)	5 (11.8)	3 (7.7)	0	10 (6.7)
State of home residence					
Arizona	12 (38.7)	9 (21.5)	7 (17.9)	13 (35.1)	41 (27.5)
Other	19 (61.3)	33 (78.5)	32 (82.1)	24 (64.9)	108 (72.5)
GGPA	3.4±0.3	3.4±0.2	3.4±0.2	3.4±0.2	3.4±0.2
NPTE	657±63.4	680.3±44.3	686.4±42.2	689.1±44.9	679.3±49.5
Licensure first-time pass rate (%)	77.4	97.6	100.0	97.3	94.0

Values are presented as number (%) or mean±standard deviation, unless otherwise stated. GGPA, grade point average at the time of graduation; NPTE, National Physical Therapy Examination.

**Table 2.** Mixed effects model associated with the National Physical Therapy Examination

Variable	UMM	Step 2		Step 3		Step 4		Step 5		Step 8		Step 9		Step 11	
		ß (SE)	t- or Z-value	ß (SE)	t- or Z-value	ß (SE)	t- or Z-value	ß (SE)	t- or Z-value	ß (SE)	t- or Z-value	ß (SE)	t- or Z-value	ß (SE)	t- or Z-value
Fixed effects															
Within-person (level 1)															
VGRE%		0.07 (0.24)	0.32	0.05 (0.24)	0.23	0.10 (0.24)	0.4	-0.05 (0.25)	-0.2	-0.07 (0.25)	-0.27	-0.41 (0.28)	-1.47	-0.32 (0.21)	-1.49
QGRE%				0.33 (0.23)	1.43	0.32 (0.23)	1.38	0.21 (0.27)	0.79	0.24 (0.27)	0.91	-0.26 (0.31)	-0.86	-0.10 (0.24)	-0.42
AGRE%						0.40 (0.22)	1.83	0.26 (0.25)	1.04	0.25 (0.26)	0.97	0.06 (0.29)	0.2	0.05 (0.22)	0.21
Average interview score								2.85 (1.92)	1.49	3.06 (1.93)	1.58	3.93 (2.17)	1.81	1.24 (1.71)	0.73
SGPA										-12.29 (23.15)	-0.53	-18.86 (24.65)	-0.77	-9.39 (19.73)	-0.48
UGPA										19.17 (26.01)	0.74	17.59 (28.63)	0.62	5.09 (19.73)	0.22
UGPA										46.09 (83.53)	0.55	76.58 (89.62)	0.86	39.00 (68.88)	0.57
Reflective essay score												-0.12 (4.09)	-0.03	-1.27 (3.12)	-0.41
GGPA														125.21 (19.19)	6.524^*^
UGPA×GGPA														40.85 (23.52)	1.74
Random effects															
Within-person (level 1)															
Residual		2,437.30 (295.16)	8.26^*,a)^	2,423.27 (294.13)	8.24^*,a)^	2,400.16 (293.01)	8.19^*,a)^	1,909.48 (267.38)	7.14^*,a)^	1,887.26 (264.27)	7.14^*,a)^	1,647.61 (274.60)	6.00^*,a)^	953.35 (159.22)	6.00^*,a)^
Intercept		78.50 (113.02)	0.70^a)^	43.00 (91.47)	0.47^a)^	4.64 (68.63)	0.07^a)^	NA	NA	NA	NA	NA	NA	NA	NA
Model fit															
Negative 2-log likelihood	1583.06	1502.78		1500.88		1497.85		1060.03		1058.84		737.64		698.4	
Akaike information criterion	1589.06	1510.78		1510.88		1509.85		1074.03		1078.84		759.64		724.4	
Bayesian information criterion	1598.07	1522.58		1525.62		1527.54		1092.41		1105.09		784.68		753.99	

UMM, unconditional means model; SE, standard error; VGRE%, verbal Graduate Record Examination rank percentile; QGRE%, quantitative Graduate Record Examination rank percentile; AGRE%, analytical Graduate Record Examination rank percentile; SGPA, precumulative science grade point average; UGPA, precumulative grade point average; GGPA, grade point average at the time of graduation; NA, not applicable.

* P<0.001.

a) Indicates Z-value.

**Table 3.** Mixed effects model associated with cumulative grade point average at the time of graduation

Variable	UMM	Step 4		Step 5		Step 6		Step 7		Step 8		Step 9	
		ß (SE)	t- or Z-value	ß (SE)	t- or Z-value	ß (SE)	t- or Z-value	ß (SE)	t- or Z-value	ß (SE)	t- or Z-value	ß (SE)	t- or Z-value
Fixed effects													
Within-person (level 1)													
VGRE%		<0.01 (0.00)	0.09	<-0.01 (0.00)	-0.47	<-0.01 (0.00)	-0.50	<-0.01 (0.00)	-0.50	<-0.01 (0.00)	-0.58	<-0.01 (0.00)	-0.47
QGRE%		<-0.1 (0.00)	-0.33	<0.01 (0.00)	0.33	<0.01 (0.00)	0.37	<0.01 (0.00)	0.71	<0.01 (0.00)	0.69	<-0.01 (0.00)	-0.33
AGRE%		<0.01 (0.00)	0.68	<-0.01 (0.00)	-0.04	<0.01 (0.00)	0.06	<0.01 (0.00)	0.22	<0.01 (0.00)	0.07	<0.01 (0.00)	0.04
Average interview score				0.02 (0.01)	2.01^*^	0.02 (0.01)	2.08^*^	0.02 (0.01)	2.31^*^	0.02 (0.01)	2.34^*^	0.02 (0.01)	2.11^*^
SGPA						0.05 (0.08)	0.61	-0.07 (0.09)	-0.7	-0.09 (0.10)	-0.91	-0.14 (0.12)	-1.22
UGPA								0.27 (0.11)	2.40^*^	0.24 (0.12)	2.12^*^	0.17 (0.14)	1.22
UGPA										0.25 (0.37)	0.66	0.37 (0.43)	0.86
Reflective essay score												0.01 (0.02)	0.53
Random effects		ß (SE)	Z-value	ß (SE)	Z-value	ß (SE)	Z-value	ß (SE)	Z-value	ß (SE)	Z-value	ß (SE)	Z-value

Within-person (level 1)													
Residual		0.05 (0.01)	8.34^**^,^a)^	0.04 (0.01)	7.14^**^,^a)^	0.0395 (0.01)	7.14^*^,^a)^	0.37 (0.01)	7.14^*^,^a)^	0.04 (0.01)	7.14^**^,^a)^	0.04 (0.01)	6.00^**^
Intercept		NA	NA	NA	NA	NA	NA	NA	NA	NA	NA	NA	NA
Model fit													
Negative 2-log likelihood	-38.88	-34.16		-39.74		-40.11		-45.73		-46.17		-32.05	
Akaike information criterion	-32.88	-22.16		-25.74		-24.11		-27.73		-26.17		-10.05	
Bayesian information criterion	-23.93	-4.55		-7.36		-3.11		-4.11		-0.08		14.99	

UMM, unconditional means model; SE, standard error; VGRE%, verbal Graduate Record Examination rank percentile; QGRE%, quantitative Graduate Record Examination rank percentile; AGRE%, analytical Graduate Record Examination rank percentile; SGPA, precumulative science grade point average; UGPA, precumulative grade point average; NA, not applicable.

* P<0.001.

** P<0.001.

a) Indicates Z-value.

## Supplementary materials

Supplement 1. Data files are available from https://doi.org/10.7910/DVN/44MVBT.

Supplement 2. Audio recording of the abstract.
